# Maternal and fetal exposures to fluoride during mid-gestation among pregnant women in northern California

**DOI:** 10.1186/s12940-020-00581-2

**Published:** 2020-04-06

**Authors:** Dawud Abduweli Uyghurturk, Dana E. Goin, Esperanza Angeles Martinez-Mier, Tracey J. Woodruff, Pamela K. DenBesten

**Affiliations:** 1 Department of Orofacial Sciences, School of Dentistry, University of California, San Francisco, San Francisco, California USA; 2 Program on Reproductive Health and the Environment, Department of Obstetrics, Gynecology, and Reproductive Sciences, University of California, San Francisco, San Francisco, California USA; 3 Department of Cariology, Operative Dentistry and Dental Public Health, Indiana University School of Dentistry, Indianapolis, Indiana USA

**Keywords:** Fluoride, Water, Pregnancy

## Abstract

**Background:**

Previous studies have shown a correlation between fluoride concentrations in urine and community water fluoride concentrations. However, there are no studies of the relationship between community water fluoridation, urine, serum, and amniotic fluid fluoride concentrations in pregnant women in the US. The aim of this study was to determine the relationship between maternal urine fluoride (MUF), maternal urine fluoride adjusted for specific gravity (MUF_SG_), maternal serum fluoride (MSF), amniotic fluid fluoride (AFF) concentrations during pregnancy, and community water fluoridation in Northern California.

**Methods:**

Archived samples of urine, serum and amniotic fluid collected from second trimester pregnant women in Northern California from 47 different communities in Northern California and one from Montana (*n* = 48), were analyzed for fluoride using an ion specific electrode following acid microdiffusion. Women’s addresses were matched to publicly reported water fluoride concentrations. We examined whether fluoride concentrations in biospecimens differed by fluoridation status of the community water, and determined the association between water fluoride concentrations and biospecimen fluoride concentrations using linear regression models adjusted for maternal age, smoking, Body Mass Index (BMI), race/ethnicity, and gestational age at sample collection.

**Results:**

Fluoride concentrations in the community water supplies ranged from 0.02 to 1.00 mg/L. MUF, MSF , and AFF concentrations were significantly higher in pregnant women living in communities adhering to the U.S. recommended water fluoride concentration (0.7 mg/L), as compared with communities with less than 0.7 mg/L fluoride in drinking water. When adjusted for maternal age, smoking status, BMI, race/ethnicity, and gestational age at sample collection, a 0.1 mg/L increase in community water fluoride concentration was positively associated with higher concentrations of MUF (B = 0.052, 95% CI:0.019,0.085), MUF_SG_ (B = 0.028, 95% CI: -0.006, 0.062), MSF (B = 0.001, 95% CI: 0.000, 0.003) and AFF (B = 0.001, 95% CI: 0.000, 0.002).

**Conclusions:**

We found universal exposure to fluoride in pregnant women and to the fetus via the amniotic fluid. Fluoride concentrations in urine, serum, and amniotic fluid from women were positively correlated to public records of community water fluoridation. Community water fluoridation remains a major source of fluoride exposure for pregnant women living in Northern California.

## Introduction

In the United States (US), water and water-based beverages contribute to approximately 75% of the total fluoride intake among adults living in communities that fluoridate their water supply [1]. The most recent estimates posted by the US Centers for Disease Control and Prevention (CDC), are that nearly three-fourths of the U.S. population with access to community water systems receive water adjusted to the federally recommended concentration of 0.7 mg/L fluoride (https://www.cdc.gov/fluoridation/statistics/2016stats.htm). This concentration of community water fluoridation in the US is higher than in Canada, where approximately one-third of Canadian communities fluoridate their water supply, and Europe, where only 3% of Europeans have fluoridated water [2, 3]. Recent studies of the potential effects of fluoride on neurodevelopment from prenatal exposures [4–8], and the onset of puberty in boys [6], suggest the need to evaluate fluoride concentrations in communities in the US and their relationship to fluoride concentrations in pregnant women.

Enamel fluorosis, which is a bioindicator for systemic fluoride exposure during times of tooth enamel formation, has been increasing in the US. In 1986–1987, 22.6% of adolescents aged 12–15 were reported to have dental fluorosis, and this increased to 40.7% in 1999–2004 [9]. In adolescents aged 16 and 17 years, fluorosis prevalence was reported to have again increased by 31.6% in 2012–2011, as compared with concentrations in 2002–2001 [10]. The purported increase in enamel fluorosis has led to concerns that overall systemic fluoride exposure is increasing, and in 2015 the CDC recommended concentrations for fluoride in drinking water be reduced from 1 mg/L fluoride to 0.7 mg/L fluoride [11]. However, the US Environmental Protection Agency has continued to allow fluoride concentrations of up to 4 mg/L in drinking water (https://www.epa.gov/ground-water-and-drinking-water/national-primary-drinking-water-regulations).

There are no contemporary measurements of systemic fluoride in adult humans in the United States, and no evaluation of their relationship to water fluoridation. Additionally, there are no contemporary studies of fluoride concentrations in pregnant women in fluoridated communities in the US, nor in fetal related tissues, despite recent concerns about effects on neurodevelopment.

To address this, we measured fluoride concentrations in urine, serum and amniotic fluid of second trimester pregnant women in Northern California. We used publicly available information on fluoride concentrations in their water systems to evaluate the relationship between reported fluoride concentrations and biomonitoring measurements, to assess the contribution of water fluoridation to measured fluoride concentrations in biological samples.

## Methods

### Study sample

Maternal urine, serum, and amniotic fluid were collected, and archived, between 2014 and 2016, from a total of 138 second trimester pregnant women with uncomplicated pregnancies from Northern California seeking care at Zuckerberg San Francisco General Hospital. Women stayed 1 to 2 days in San Francisco, which is fluoridated, and fasting urine samples were collected on day 2. The specific gravity of the urine samples was measured at the time of collection. Samples were labeled with a unique identification and barcode and then aliquoted into smaller Cryovials® and stored at -80 °C. Of these samples, 50 were selected from boxes whose records were easily accessible, with an equal number selected from sample collection dates in 2014, 2015, and 2016. The samples were selected from women residing in different communities across Northern California, and one residing in Montana. Two of the samples were excluded from analysis due to the unavailability of water fluoride concentrations. One sample was further excluded from amniotic fluid fluoride analysis due to sample unavailability, leaving a final study population of 48 for urine and maternal serum analysis and 47 for those with amniotic fluid. Women’s addresses were abstracted from the medical record. We were only able to obtain zip codes and not full addresses for four women.

The women were racially and ethnically diverse, with an average age of 25.7 years (Table 1). The majority had a high school education or less, and more than a third had no children at the time of sample collection (Table 1). More than two-thirds of the participants reported smoking in the past year. This study was approved by the University of California, San Francisco Committee on Human Research.

**Table 1:** Descriptive statistics

	Mean (SD)
Fluoride concentrations	
Water fluoride (mg/L)	0.50 (0.33)
Maternal urine fluoride (mg/L)	0.63 (0.38)
Maternal urine fluoride adjusted for SG (mg/L)	0.63 (0.35)
Maternal serum fluoride (mg/L)	0.016 (0.014)
Amniotic fluid fluoride (mg/L)	0.017 (0.011)
Gestational age at sample collection	20.5 (2.1)
Demographics	
Age	25.7 (4.9)
BMI	28.0 (6.2)
	N (%)
Race/ethnicity	
Latina	9 (18.8)
Black	12 (25.0)
White	20 (41.7)
Asian/Pacific Islander	7 (14.6)
Educational attainment	
Less than high school	3 (6.3)
High school/GED	21 (43.8)
Some college	17 (35.4)
College grad or postgrad	6 (12.5)
Missing	1 (2.1)
Smoked in the past year	
No	14 (29.2)
Yes	34 (70.8)

### Fluoride measurements

Fluoride concentrations were determined using a modification of the hexamethyldisiloxane (HMDS; Sigma Chemical Co.) microdiffusion procedure of Taves [12], as modified by Martínez- Mier et al. (Martinez-Mier et al., 2011). Fluoride concentrations were determined by comparing the millivolt reading of each sample to standard curves, covering the range of the samples’ values, prepared from the data for standard solutions of diffused fluoride measured at the time the samples were analyzed. In neutral solutions, the limit of detection of the ion specific fluoride electrode is 0.02 mg/L fluoride. The precision and validity of the modified microdiffusion technique used in our analysis have been reported elsewhere (Martinez-Mier et al., 2011). Fluoride concentrations in urine were measured, and were also adjusted for specific gravity using the Levine Fahy equation: [Concentration_SG normalized_ = Concentration_specimen_ (SG_reference_ – 1)/(SG_specimen_ – 1)] [13]. SG_reference_ is the median SG for the cohort , with one sample that had a specific gravity of 1 excluded. Average water fluoride concentrations in the communities in which women lived at the year in which the samples were collected were obtained from the California Water Board website https://www.waterboards.ca.gov/drinking_water/certlic/drinkingwater/Fluoridation.html, or from the water quality report of the individual city of residence using the participant addresses.

### Statistical analyses

We evaluated the correlation between biological measures of fluoride and community fluoride concentrations using Pearson’s correlation. MSF, MUF, MUFSG, and AFF concentrations were log-transformed to make the distribution approximately normal. We used linear regression to evaluate the relationship between each of the biological measures of fluoride and concentrations of fluoride in community water. We controlled for the following potential confounders: maternal age [14, 15] smoking status [16, 17], BMI [18], gestational age at the time of sample collection, and race/ethnicity.

The regression model took the form *Y = β*_*0*_ *+ β*_*1*_*X*_*1*_ *+ β*_*2*_*X*_*2*_ *+ β*_*3*_*X*_*3*_ *+ β*_*4*_*X*_*4*_ *+ β*_*5*_*X*_*5*_ *+ β*_*6*_*X*_*6*_ *+ ɛ*, where *Y* is the dependent variable (the measures of fluoride in each of the biospecimens); *X*_*1*_ is community water fluoride concentration, *X*_*2*_ is maternal age; *X*_*3*_ is a maternal smoking status (coded as 0 if the subject reported not smoking and 1 if the subject reported smoking in the past year); *X*_*4*_ is maternal BMI at the time of sample collection, *X*_*5*_ is reported maternal race/ethnicity, *X*_*6*_ is the gestational age at the time of sample collection, and ε is the individual-specific error.

We then used Welch’s t-tests to compare biological measurements of fluoride between communities with water fluoridated in accordance with US federal recommendations (= > 0.7 mg/L) and communities with less fluoridated water (< 0.7 mg/L). Three samples were associated with community water fluoride concentrations of 0.69 mg/L, and these values were rounded up and included in the group with fluoridated water adhering to federal recommendations (= > 0.7 mg/L).

To compare our results with urine fluoride concentrations of Canadian subjects [3] in communities with high and low fluoride concentrations, we reanalyzed our data using the fluoride concentrations similar to those in the Canadian study. In this study, fluoridated communities were defined as those with 0.3 mg/L or greater fluoride in water, and non-fluoridated communities had less than 0.3 mg/L fluoride in water.

All data analyses were conducted using R version 3.6.0.

## Results

**Fig. 1:**
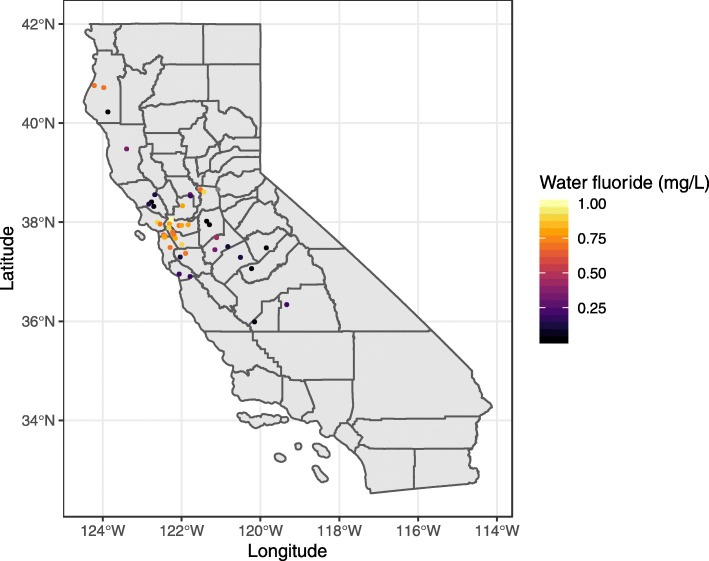
Map of the location of pregnant women participants and the community water fluoridation concentrations. Note: One participant lived in Montana at the time of sample collection but was excluded from this map for clarity

Participants primarily resided in communities across Northern and Central California, and fluoride concentrations recorded for the community water samples ranged from 0.02 to 1.00 mg/L (Fig. 1). One participant lived in Montana but is not shown in Fig. 1 for map clarity. Following acid diffusion, fluoride could be measured within the linear range of the fluoride electrode in all patient samples. MSF and AFF concentrations were similar to one another, but an order of magnitude lower than the concentrations observed in community water and MUF concentrations (Table 1).

**Fig. 2:**
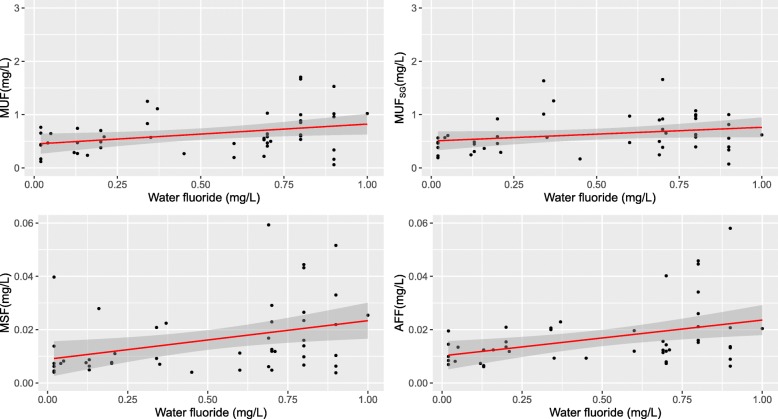
Scatter plots of community water fluoride concentrations with fluoride concentrations in maternal urine, maternal urine adjusted for specific gravity, maternal serum, and amniotic fluid

Mean MUF concentration, with and without adjustment for specific gravity (SG) in the communities with = > 0.7 mg/L, were similar to the mean water fluoride concentration, whereas in communities with less than 0.7 mg/L fluoride, MUF and MUF_SG_ concentrations were higher than the mean community water fluoride concentrations (see Table 2). While there is a clear correlation between MUF and community water fluoridation, there is also much variability in the individual MUF, particularly as fluoride concentrations in the drinking water increase (Fig. 2 and Table 3).

**Table 2:** Concentrations of fluoride in maternal urine, serum, and amniotic fluid by community water fluoridation concentration

	Fluoridation below recommended concentrations(< 0.7 mg/L)		Fluoridation in accordance with federal recommendations (> = 0.7 mg/L)		
N	24		24		
Fluoride concentrations	Mean ± SD	Min, Max	Mean ± SD		Min, Max
Community water	0.20 ± 0.18	0.02, 0.60	0.80 ± 0.092	*	0.69, 1.00
Maternal urine	0.52 ± 0.28	0.12, 1.25	0.74 ± 0.44	*	0.060, 1.70
Maternal urine adjusted for specific gravity	0.57± 0.35	0.17, 1.63	0.69 ± 0.34^#^		0.073, 1.66
Maternal serum	0.011 ± 0.0086	0.0040, 0.040	0.021 ± 0.015	*	0.0038, 0.059
Amniotic fluid	0.013 ± 0.0052	0.0061, 0.023	0.021 ± 0.014^#^	*	0.0063, 0.058

*The high fluoridation mean concentration is significantly different from the low fluoridation mean concentration at the 0.05 concentration using Welch’s t-test

^#^*N* = 23

**Table 3:** Correlation matrix between log-transformed fluoride concentrations in maternal urine, maternal urine adjusted for specific gravity, maternal serum, and amniotic fluid with fluoride concentrations in community water.

Fluoride concentrations	Community water	Maternal urine	Maternal urine adjusted for specific gravity	Maternal serum	Amniotic fluid
Community water	1.00	0.22	0.21	0.39**	0.41**
Maternal urine	0.22	1.00	0.80***	0.38**	0.28
Maternal urine adjusted for specific gravity	0.21	0.80***	1.00	0.41**	0.37*
Maternal serum	0.39**	0.38**	0.30	1.00	0.52***
Amniotic fluid	0.41**	0.28	0.37*	0.52***	1.00

Note: The maternal urine adjusted for specific gravity correlations are based on 47 individuals. One person had a urinary specific gravity of 1, which corresponds to a specific-gravity adjusted fluoride concentration of 0. This is undefined when log-transformed and therefore cannot be included in the computation of correlations. We log-transformed the fluoride concentrations in maternal urine, maternal urine adjusted for specific gravity, maternal serum, and amniotic fluid to make the distributions approximately normal. The community water distribution was not transformed as it was not skewed. *p=0.05 **p=0.01 ***p=0.001

A 0.1 mg/L increase in the community water fluoride concentration was associated with an increase of 0.052 (95% CI: 0.019, 0.085) in MUF; and of 0.028 (95% CI: 0.006, 0.062) in MUF_SG_ after adjusting for covariates (Table 4). The associations between community water fluoride concentrations and MSF and AFF were very similar: a 0.1 mg/L increase in community water fluoride was associated with increases of 0.001 (95% CI 0.000, 0.003) in MSF and 0.001 (95% CI 0.000, 0.002) AFF. The unadjusted associations were strongest between water fluoride and AFF (Table 3). For all biospecimens, we did not find any effect of the potential co-founders, including smoking, which in adolescents has been reported to be associated with increased plasma fluoride concentrations [19].

**Table 4:** Adjusted and unadjusted linear associations of 0.1 mg/L increase in community water fluoride concentrations with fluoride concentrations in maternal urine, serum, and amniotic fluid

	Relationship with community water fluoride	
Fluoride concentrations	Unadjusted (95% CI)	Adjusted^a^ (95% CI)
Maternal urine	0.037 (0.006, 0.069)	0.052 (0.019, 0.085)
Maternal urine adjusted for specific gravity	0.026 (-0.004, 0.057)	0.028 (-0.006, 0.062)
Maternal serum	0.001 (0.000, 0.003)	0.001 (0.000, 0.003)
Amniotic fluid	0.001 (0.000, 0.002)	0.001 (0.000, 0.002)

^a^ Adjusted for maternal age, smoking status, *BMI* race/ethnicity, and gestational age at sample collection. Likelihood ratio tests indicated no significant differences between the adjusted and non adjusted models

In a previous study of community water fluoride concentrations and associated MUF_SG_ in Canada, [3], fluoridated communities had fluoride concentrations greater than 0.3 mg/L fluoride in drinking water, and non-fluoridated communities had 0.3 mg/L fluoride or less in drinking water. To compare our results with those found in the Canadian study, we compared MUF_SG_ concentrations of pregnant women across communities with less than or greater than 0.3 mg/L fluoride in drinking water. We found the mean urine fluoride concentrations were similar in our Northern California sample to those in Canada (Table 5).

**Table 5:** Comparison of fluoride concentrations in mg/L across communities with low and high fluoridation concentrations in Northern California and Canada

	Community water fluoride <=0.3 mg/L (Mean ± SD)		Commuunity water fluoride (> 0.3 mg/L) (Mean ± SD)		
Fluoride concentrations	Canada	Northern California (*N* = 17)	Canada	Northern California (*N* = 31)	
Water	0.12 ± 0.06	0.099 ± 0.076	0.61 ± 0.11	0.71 ± 0.18	*
Maternal urine		0.46 ± 0.20		0.72 ± 0.43	*
Maternal urine adjusted for specific gravity	0.41 ± 0.28	0.45 ± 0.18	0.71 ± 0.38	0.74 ± 0.38	*

* The Northern California high fluoridation mean concentration is significantly different from the Northern California low fluoridation mean concentration at the 0.01 confidence level using Welch’s t-test

## Discussion

We measured fluoride concentrations in urine, serum, and amniotic fluid from 47 second trimester pregnant women primarily living in Northern California between 2014 and 2016. To our knowledge, this is the first time these types of data have been reported in the US. In our study, which used archived biological samples, community water fluoride samples from the time of collection were not available. Therefore, we used public records to determine the water fluoride concentrations for each community in the year that the samples were collected.

Fluoride measurements using a fluoride ion specific electrode are highly specific when the sample is buffered to a pH below 7 to prevent the interference of hydroxyl ions. However, fluoride concentrations in serum or plasma are close to the limit of detection (LOD) of the electrode of 0.02 mg/L (mg/L) and therefore the hexamethyldisiloxane (HMDS) facilitated diffusion method, originally derived by Taves [12], and further modified by Martinez-Mier et al., (Martinez-Mier et al., 2011) quantitatively transfers fluoride from the sample into an alkaline trapping solution of smaller volume. This process results in fluoride concentrations in the solution that are above the LOD and on the linear portion of the standard curve. Furthermore, this method is preferred for samples that contain protein as it also releases additional fluoride ions that may be bound to proteins through binding to cations or other positively charged molecular groups.

Urine from healthy individuals, which has a relatively low protein content, can be measured directly, without diffusion. However, because we did not know the medical history of our sample population, we used the diffusion method to measure the fluoride concentration in urine, as well in serum and amniotic fluid. Urine fluoride concentrations were measured in spot samples rather than 24-hour urine samples. Urine spot samples have been shown to be an accurate assessment of fluoride ingestion on a population basis [20]. We found similar MUF_SG_ concentrations for pregnant women in Northern California relative to their community water fluoride concentrations, as reported by Till et al. in a Canadian population [3]. Community water fluoride concentration and MUF_SG_ were associated in both this study, and the study by Till et al., although in this study the confidence intervals for both the adjusted and unadjusted associations crossed the null.

The formula used to correct for specific gravity was originally generated in 1945 by Levine and Jahy [13], to adjust for urinary lead concentrations. However, this formula has recently been questioned; in particular, there are concerns about whether it overcompensates for the confounding effect of specific gravity in the absence of an appropriately weighted exponential adjustment factor for the substance of interest [13, 21, 22]. There is no such factor yet defined for fluoride; therefore, we also present associations with maternal urine unadjusted for specific gravity (Table 2). We found that unadjusted maternal urine fluoride was significantly positively associated with water fluoride concentrations.

The similarity in urine fluoride measures in Till’s study of a larger cohort of Canadian women [3] and ours, supports the validity of our relatively small sample size, and underlines the usefulness of MUF as a biomarker to compare study outcomes relative to fluoride intake. However, we did not have access to information on additional possible fluoride exposure through dental products, or the use of tea, bottled drinks or water; which is a limitation to this study. With the inclusion of this data, we may have identified more differences between our US derived samples and those from the Canadian study.

Similarly to the findings by Smith et al. [23] and Zipkin et al. [20] in the US in the 1950s, we found that the mean concentration of fluoride in urine of women from fluoridated communities (MUF) was similar (0.74 mg/L) to mean concentrations of fluoride in drinking water (0.8 mg/L) (see Table 2). However, in our study, mean urine fluoride concentrations from communities with lower water fluoride concentrations (0.52 mg/L) were more than twice that of community water fluoride concentrations (0.2 mg/L). It is possible that the relative increase in urine fluoride concentrations of women from non-fluoridated communities, was due to the overnight stay in fluoridated San Francisco. However, these values for non-fluoridated communities were similar to those reported by Till et al. [3], suggesting that in both Canada and the US, there is increased exposure to other sources of fluoride outside of the community water supply. For example, consumption of bottled drinks made in areas with higher fluoride concentrations, which are then consumed in the lower fluoride areas, may increase fluoride exposure. This so-called “halo” effect of fluoride exposure [24, 25] would not have been present in previous times when most community water, including that used for the manufacturing of food and beverages, was at concentrations less than 1.0 mg/L fluoride.

Smith et al. previously reported that the fold increase of urine fluoride as compared to blood fluoride concentration (4.3 fold), was lower in communities with low fluoride in drinking water as compared to communities with higher water fluoride (28 fold) [23]. This reported difference between lower and higher fluoride exposure suggests an increase in glomerular filtration rate with increasing fluoride exposure. However, Malin et al. [26] showed evidence of reduced glomerular filtration rates associated with increased water fluoride, which would result in a decrease, rather than an increase in the fold difference between urine and blood fluoride [27]. Instead, the relatively high blood fluoride concentrations as compared to urine fluoride in the low fluoride group reported by Smith et al., may have been due to sampling techniques as suggested by Taves [28], or more likely, because they had reached the limits of detection in their method for measuring low concentrations of fluoride in blood. Our results show a consistent and significant association between water fluoride, urine fluoride and serum fluoride, and support the use of urine fluoride as a biomarker for systemic fluoride exposure.

We found fluoride concentrations in amniotic fluid to be similar to maternal serum fluoride concentrations, and both were positively correlated to community water fluoride concentrations. These concentrations of amniotic fluid fluoride are similar to those reported by Ron et al. [29] drawn during mid trimester amniocentesis. However, in that study, maternal plasma concentrations were higher than what we measured for maternal serum. Fluoride concentrations in plasma and serum are comparable, and therefore a possible reason for the differences between ours and Ron’s study, may be related to their use of direct fluoride measurements, without prior diffusion, which would therefore be at the limits of fluoride detection by the electrode.

Our finding of similar fluoride concentrations between maternal serum and amniotic fluid supports direct diffusion of fluoride from maternal serum, without a placental barrier. This is supported by data from Amstrong [30] who found that ashed sera from maternal and umbilical blood obtained after cesarean section, contained similar concentrations of fluoride. Shen and Taves subsequently measured fluoride in maternal and cord blood at birth in 5 subjects using the fluoride diffusion method and found the concentration of fluoride in cord blood to be approximately 75% of maternal serum. They concluded that the high positive correlation between maternal and cord blood (0.86) showed that fluoride passively diffuses across the placenta [31]. It is likely that the difference between these studies may have been related to the time of sampling, as circulating fetal fluoride concentrations are reduced later in gestation as fluoride is taken up into the rapidly growing skeleton. Though Gedalia is frequently quoted as providing evidence that the placenta creates a barrier to fluoride [32] he reversed this in a later publication [33] confirming the free passage of fluoride between mother and fetus. The difference in his findings over time has been attributed to methodological differences in his fluoride analysis.

Gupta et al. reported that when maternal plasma fluoride concentrations were greater than 0.4 mg/L, that fetal cord blood fluoride concentrations were relatively reduced [34]. This finding has been interpreted as evidence that a placental barrier occurs at high fluoride concentrations. However, 0.4 mg/L is an extremely high plasma fluoride concentration, and is over 10 fold the highest maternal serum concentration measured in our study. Reports of these high plasma fluoride concentrations suggest the possibility that there were measurement errors. If they are correct, then is likely that other systemic toxic effects that would occur at these concentrations [35], would account for the differences in fluoride concentrations, rather than a specific placental barrier to fluoride ion diffusion.

The early formed amniotic fluid is formed by diffusion of maternal plasma through the placenta, and is replaced by fetal urine at about 20 weeks. This early formed amniotic fluid is contained in the neural tube when it closes at approximately the 4th week of pregnancy, [36], and forms the nascent cerebrospinal fluid (CSF) [37], where it interacts with the developing brain. The importance of this early cerebral spinal fluid in brain development is demonstrated in Xenopus embryos, where exposure to a mixture of ubiquitous chemicals at concentrations found in human amniotic fluid affect thyroid hormone-dependent transcription, gene expression, brain development and behaviour in early embryogenesis [38]. While our analyses were done on midtrimester amniotic fluid, given recent population based correlations between systemic fluoride exposure and neurotoxicity [8], further studies of how fluoride may also affect early brain development in concert with other environmental stressors, are warranted.

## Conclusions

This study adds evidence in support of the use of urinary fluoride as a biomarker for systemic fluoride exposure in pregnant women. However, as the association between maternal serum fluoride and water fluoride was highly significant, maternal serum maybe the best proxy for fetal exposure in studies of the impact of fluoride exposure on fetal and infant development. When maternal serum fluoride is not available, then urine fluoride is also a good proxy for fetal exposure.

## Data Availability

The datasets generated during and/or analysed during the current study are not publicly available due to patient privacy protections but are available from the corresponding author on reasonable request.

## Abbreviations

AFF: Amniotic fluid fluoride
BMI: Body mass index
CDC: Centers for Disease Control and Prevention
CSF: Cerebrospinal fluid
HMDS: Hexamethyldisiloxane
IQ: Intelligence quotient
LOD: Limit of detection
MSF: Maternal serum fluoride MUF_SG_ maternal urinary fluoride corrected for specific gravity
MUF: Maternal urinary fluoride
SG: Specific gravity
US: United States
