# Correction: Maternal and fetal exposures to fluoride during mid-gestation among pregnant women in northern California

**DOI:** 10.1186/s12940-025-01222-2

**Published:** 2025-10-09

**Authors:** Dawud Abduweli Uyghurturk, Dana E. Goin, Esperanza Angeles Martinez-Mier, Tracey J. Woodruff, Pamela K. DenBesten

**Affiliations:** 1 Department of Orofacial Sciences, School of Dentistry, University of California, San Francisco, San Francisco, CA USA; 2 Program on Reproductive Health and the Environment, Department of Obstetrics, Gynecology, and Reproductive Sciences, University of California, San Francisco, San Francisco, CA USA; 3 Department of Cariology, Operative Dentistry and Dental Public Health, Indiana University School of Dentistry, Indianapolis, IN USA

**Correction: Environ Health 19, 38 (2020)**

**https://doi.org/10.1186/s12940-020-00581-2**

1) In Table 3, the **correlation of 0.30 between MUF(SG) and MSF should be 0.41 (significant at *p*** < **0.01)**. 

2) The results presented in the statement “a 0.1 mg/L increase in community water fluoride was associated with increases of 0.001 (95% CI 0.000, 0.003) in MSF and 0.001 (95% CI 0.000, 0.002) AFF”, were rounded to 3 significant digits and the confidence intervals had a lower bound of zero. When rounded to 4 digits, the relationship between a 0.1 mg/L increase in water fluoride and MSF is 0.0014 (95% CI 0.0002, 0.0026) and the relationship between water fluoride and AFF is 0.0010 (95% CI 0.0001, 0.0019).

3) The lower limit for the 95% CI for MUF(SG) in Table 4 of −0.006 is correct, while the stated lower limit of + 0.006 as appears in the text, is a typo, and should reflect the **− 0.006** value in the table.

The original article has been updated.

**Reference**

1. Abduweli Uyghurturk D, Goin DE, Martinez-Mier EA, et al. Maternal and fetal exposures to fluoride during mid-gestation among pregnant women in Northern California. Environ Health. 2020;19:38. 10.1186/s12940-020-00581-2.

